# MMP-9 and IL-1β as Targets for Diatoxanthin and Related Microalgal Pigments: Potential Chemopreventive and Photoprotective Agents

**DOI:** 10.3390/md19070354

**Published:** 2021-06-22

**Authors:** Luigi Pistelli, Clementina Sansone, Arianna Smerilli, Marco Festa, Douglas M. Noonan, Adriana Albini, Christophe Brunet

**Affiliations:** 1 Stazione Zoologica Anton Dohrn, Istituto Nazionale di Biologia, Ecologia e Biotecnologie Marine, Villa Comunale, 80121 Napoli, Italy; luigi.pistelli@szn.it (L.P.); arianna.smerilli@szn.it (A.S.); christophe.brunet@szn.it (C.B.); 2 Laboratory of Vascular Biology and Angiogenesis, IRCCS MultiMedica, 20138 Milan, Italy; marcomariogiacomo.festa@multimedica.it (M.F.); adriana.albini@multimedica.it (A.A.); 3 Department of Biotechnology and Life Sciences, University of Insubria, 21100 Varese, Italy; douglas.noonan@uninsubria.it; 4 Unit of Molecular Pathology, Biochemistry and Immunology, IRCCS MultiMedica, 20138 Milan, Italy

**Keywords:** diatoxanthin, alloxanthin, oxidative stress, carotenoids, anti-inflammation, ROS, photoprotection, chemoprevention, metalloproteinase MMP-9, interleukin 1 beta

## Abstract

Photochemoprevention can be a valuable approach to counteract the damaging effects of environmental stressors (e.g., UV radiations) on the skin. Pigments are bioactive molecules, greatly attractive for biotechnological purposes, and with promising applications for human health. In this context, marine microalgae are a valuable alternative and eco-sustainable source of pigments that still need to be taken advantage of. In this study, a comparative in vitro photochemopreventive effects of twenty marine pigments on carcinogenic melanoma model cell B16F0 from UV-induced injury was setup. Pigment modulation of the intracellular reactive oxygen species (ROS) concentration and extracellular release of nitric oxide (NO) was investigated. At the cell signaling level, interleukin 1-β (IL-1β) and matrix metallopeptidase 9 protein (MMP-9) protein expression was examined. These processes are known to be involved in the signaling pathway, from UV stress to cancer induction. Diatoxanthin resulted the best performing pigment in lowering MMP-9 levels and was able to strongly lower IL-1β. This study highlights the pronounced bioactivity of the exclusively aquatic carotenoid diatoxanthin, among the others. It is suggested increasing research efforts on this molecule, emphasizing that a deeper integration of plant ecophysiological studies into a biotechnological context could improve the exploration and exploitation of bioactive natural products.

## 1. Introduction

Skin represents a barrier between organism and environment, being exposed to direct oxidative injury determined by ultraviolet (UV) radiation, representing the first level of immune defense. An acute UV exposure causes short- and long-term deleterious effects on human epidermis, such as sunburn, connective tissue deterioration, DNA injury, and immune suppression [1]. In addition, chronic exposure to UV radiation can be deleterious, leading to various long-term damaging effects, including skin aging (photoaging) or skin cancer (photocarcinogenesis). Photoaging results from a cumulative damage caused by UV exposure-induced oxidative products [2]. Indeed, UV-radiation induces in skin tissue an increased generation of intracellular reactive oxygen species (ROS), an over-expression of matrix metalloproteinases (MMPs), and the production of proinflammatory cytokines (Interleukins-IL) through extracellular nitric oxide (NO) signaling [3]. An increase of intracellular ROS induces the phosphorylation of protein kinases through mitogen-activated protein kinases (MAPK) signaling pathway which directly promotes the phosphorylation of the activator protein-1 (AP-1) complex, thus up-regulating the expression of MMPs [4]. MMPs signaling is responsible for degrading the extracellular matrix (ECM) proteins, such as collagen, fibronectin, elastin, and proteoglycans. This degradation of the skin’s ECM is the main cause for UV irradiation-induced photoaging [5]. MMP-9, one of the main primary UV-inducible collagenolytic enzymes, is known to degrade collagen type IV in fragments, and to play an important role in tumor invasion and angiogenesis by enhancing not only migration capabilities but also the availability of vascular endothelial growth factor (VEGF) in malignant tumors [1]. Moreover, MMP9 allows invasion of basal cell carcinoma (BCC) and squamous cell carcinoma (SCC), and it is related to the radial growth phase (RGP) of melanoma carcinogenesis [1]. It is known that intracellular signaling pathway triggered by ROS is of crucial importance in photocarcinogenesis and in the metastatic tumor processes [6,7]. Therefore, compounds able to inhibit ROS generation and MMPs downstream activation may act as photo-chemopreventive agents against UV-induced photoaging. In this context, natural bioactive compounds from photosynthetic organisms, such as higher plants, micro- or macro-algae, are possible ingredients for skin protection medicaments [2]. Among the phytochemicals involved in light energy-related biological processes—from photosynthesis to vision—pigments are represented by different families, such as chlorophylls, carotenoids, or phycobiliproteins [8,9,10]. Most pigments are highly bioactive, and so they can act on living tissue or cells. This characteristic explains their numerous applications [11,12], being employed besides as dyestuff, as food and animal feed additives [10,13,14]. A few carotenoids have been recognized for their health-benefits, among them β-carotene and lycopene [15], while more than eight hundred of carotenoids have been described so far [9]. Although many pigments are shared by both terrestrial and aquatic organisms, some of them are exclusively aquatic, like chlorophylls c and phycobiliproteins. In addition, many carotenoids (e.g., fucoxanthin, peridinin, diadinoxanthin, siphonein, etc.) are exclusively aquatic pigments. The differences between terrestrial and aquatic systems molecular modifications are probably related to the peculiar underwater light spectrum [16], compared to the terrestrial light spectrum. Macroalgae and microalgae inhabiting aquatic environments also display different pigment profiles. Among them, eukaryotic microalgae due to their complex evolution [17] present a marked chemodiversity between taxonomic groups [18]. Intriguingly, along with the microalgal evolution and diversification, a decreased number of the main carotenoids can be noted, from at least five major compounds in chlorophytes (violaxanthin, lutein, neoxanthin, antheraxanthin, α- and/or β-carotene) to three in diatoms (fucoxanthin, diadinoxanthin, and β-carotene) [19]. In addition, the so-called photoprotective xanthophyll cycle (XC) involves three xanthophylls (violaxanthin, antheraxanthin and zeaxanthin) in terrestrial plants or green and brown algae, and only two (diadinoxanthin and diatoxanthin) in the more recent microalgal groups (Bacillariophyceae, Xanthophyceae, Haptophyceae, or Dinophyceae [20]).

This study aims to seek pigments with a photochemopreventive activity, focusing on microalgal compounds for biomedical and cosmeceutical future applications. Indeed, microalgae are recognized as an emergent eco-sustainable resource, being considered as suitable cell factories for the production of many natural bioactive substances [21,22,23]. A biological activity exploration of twenty pigments was conducted starting from a selection based on the exclusive—or not exclusive—presence in microalgal groups (Table 1). The ten pigments belonging to the category “aquatic and terrestrial” were selected based on their potential or known bioactive interest (e.g., lutein, zeaxanthin, neoxanthin, β-carotene, amongst others; Table 1). The ten “aquatic specific” pigments targeted in this study were selected based on (i) the lack of bioactivity information, (ii) a similar role covered by some of these pigments in microalgae with pigments selected in the other group (e.g., photoprotective role), (iii) previous information on their concentration variations in function of the light environment perceived by microalgae [20,24,25,26,27]; and (iv) pigments from more recent (at an evolutive point of view) microalgal groups (e.g., from diatoms, dinophytes, or haptophytes) versus pigments from older microalgal groups (cyanophytes, chlorophytes).

After a comparative analysis of the antioxidant activity of pigments, their toxicity was tested on two cell lines: B16-F0 and HaCaT. The latter is a keratinocyte cell line from adult human skin widely used in research because of its high capacity to differentiate and proliferate in vitro [28]. In order to evaluate the photochemopreventive activity of pigments the parental skin melanoma B16-F0 cell line, isolated from C57BL/6 mice, was selected being a well-known model for cellular differentiation studies [29]. Algal pigment bioactivity assessment focused on photoaging and inflammatory processes that promote skin cell migration and invasion by upregulating MMP-9 [30], through the modulation of intracellular signaling pathway triggered by ROS towards the pro-inflammatory release of NO and Interleukin 1β (IL-1β).

## 2. Results

### 2.1. DPPH (2,2-Diphenyl-1-picrylhydrazyl) Scavenging

DPPH assay was performed to compare the scavenging activity of the twenty pigments.

A great variability of DPPH scavenging, ranging from 0 to 73%, was revealed amongst pigment species and over the range of tested concentrations (Table 2).

Some pigments, like 19′-hexanoyloxyfucoxanthin, 19′-butanoyloxyfucoxanthin, antheraxanthin, lycopene, chlorophyll c_3_, zeaxanthin, diadinoxanthin, peridinin, violaxanthin, and alloxanthin, displayed a DPPH scavenging in a dose dependent response (Table 2). Conversely, diatoxanthin presented a reverse trend (Table 2), with a decrease of scavenging potential from low (47%) to high concentration (20%). At the lowest pigment concentration (25 ng mL^−1^), lutein, diatoxanthin, 19′-butanoyloxyfucoxanthin, and α-carotene displayed significantly greater DPPH scavenging activity than the other pigments tested (at least *p* < 0.05).

Due to the capacity of some pigments to significantly counteract ROS generation at low concentration (25 ng mL^−1^), the in vitro experiments on the B16-F0 cell line were carried out applying each pigment at this pre-assessed concentration, as well as for the interests in natural bioactive compounds able to exert positive effects and modulating cellular processes at low concentration.

### 2.2. Pigment Cytotoxicity on B16-F0 and HaCaT Cells and Protective Effect on B16-F0 Cells from UV Radiations

Viability tests were conducted to both assess the potential toxicity of the pigments on the biological system B16-F0 (before UV exposure), as well as to investigate the potential viability recovery induced by pigments after UV exposure.

**Figure 1:**
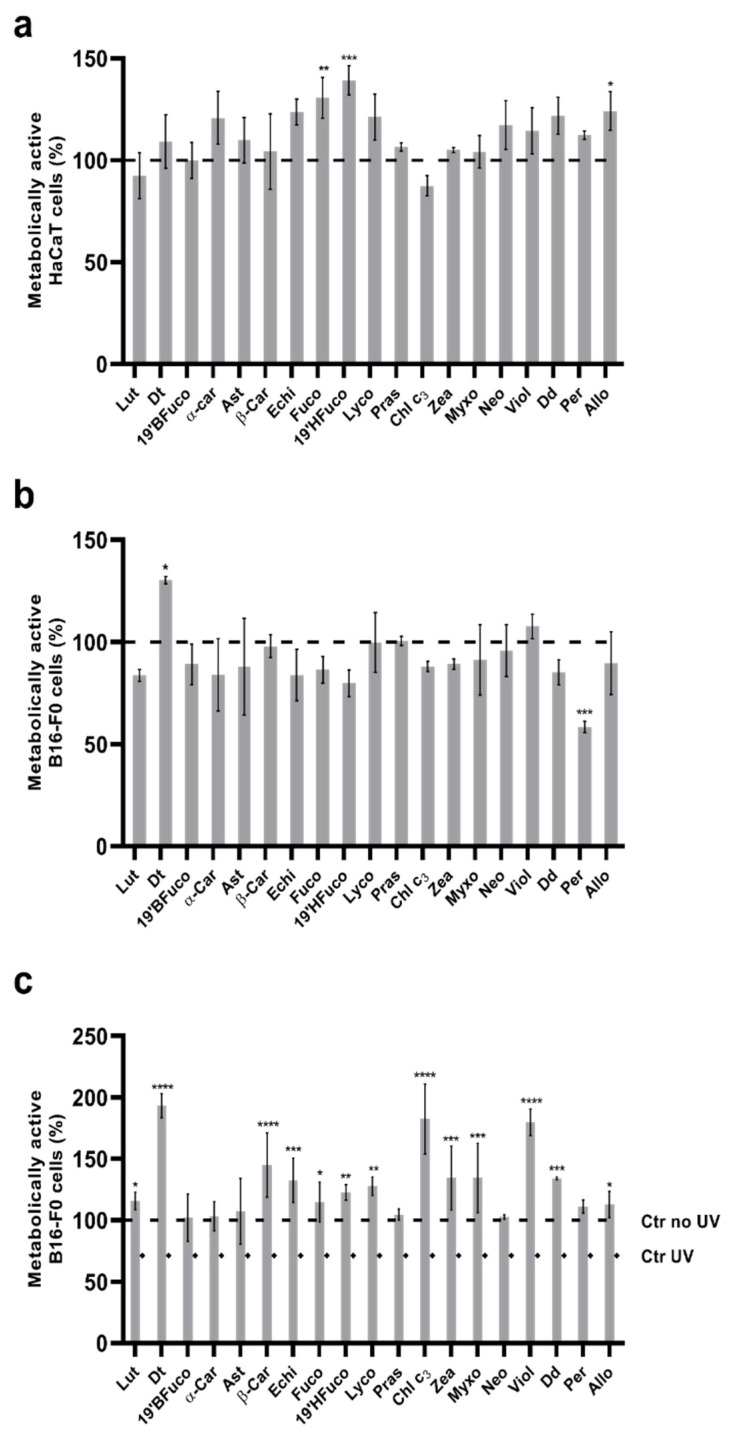
MTT (3-[4,5-dimethylthiazol-2-yl]-2,5-diphenyltetrazolium bromide) cell viability assays on HaCaT and B16-F0 cells. (**a**) Relative cytotoxicity of pigments on HaCaT cells. Values are expressed as mean ± SD of metabolically active cells compared to control (no pigments, 100% of metabolically active cells). (**b**) Relative cytotoxicity of pigments on B16-F0 cells. Values are expressed as mean ± SD of metabolically active cells compared to control (no pigments, 100% of metabolically active cells). (**c**) Protective effect of pigments on B16-F0 cells from UV radiation. Values are expressed as mean ± SD of metabolically active cells compared to control (no pigments with UV exposure, 71% of metabolically active cells). Asterisks indicate the statistically significant difference compared to the respective control (**** *p* ≤ 0.0001, *** *p* ≤ 0.001, ** *p* ≤ 0.01, * *p* ≤ 0.05; Dunnett’s test). Ctr: control.

Both the B16-F0 and HaCaT cell proliferation was not affected by pigment addition (at the 25 ng mL^−1^ concentration; *p* > 0.05), except for B16-F0 cells treated with peridinin (58% cell viability; *p* < 0.001; Figure 1a,b). Conversely, diatoxanthin induced an increase of the percentage of metabolically active cells compared to the control without pigment (*p* < 0.05) in B16-F0 cells, while fucoxanthin, 19′-hex-fucoxanthin, and alloxanthin significantly increased the percentage of metabolically active HaCaT cells by 20 to 40% (Figure 1b; at least *p* < 0.05).

UV radiations induced a significant decrease (*p* < 0.05) of metabolically active cells compared to the control (no UV; Figure 1c). All pigments exerted a significant protective effect (Figure 1b), resulting in the enhancement of metabolically active cells respect to the cell line exposed to UV radiations without pigment pre-treatment (at least *p* < 0.05). Some pigments greatly enhanced the percent of metabolically active cells, which reached a concentration even higher than in the control without UV exposure: diatoxanthin, violaxanthin, chlorophyll c_3_ (*p* < 0.0001) and β-carotene, echinenone, and diadinoxanthin (*p* < 0.001).

### 2.3. Intracellular Reactive Oxygen Species (ROS) Concentration

**Figure 2:**
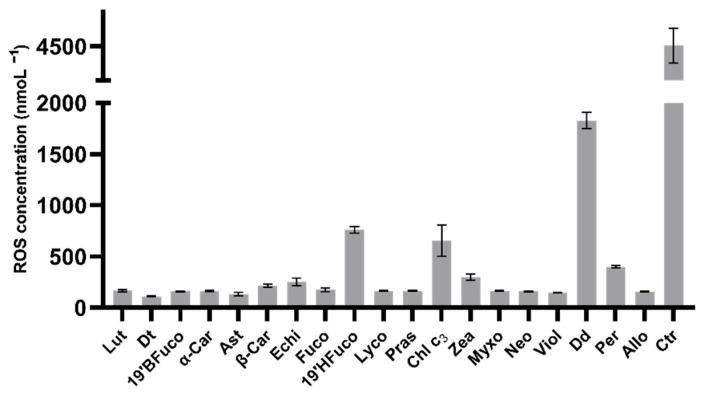
Intracellular ROS measurements.

ROS measurements were carried out to assess the UV injury on the B16-F0 cells and to investigate the amplitude of pigments in protecting cells against UV damages. UV radiations induced a strong production of intracellular ROS, reaching 4500 nmol L^−1^ (Figure 2). All pigments were able to scavenge ROS, significantly decreasing their concentration (*p* < 0.0001; Figure 2), in agreement with the previous results on pigments’ capacity to modulate the percent of metabolically active cells. Not all pigments displayed the same capacity for ROS counteracting. Low performing pigments were chlorophyll c_3_, 19′-hexanoyloxyfucoxanthin and diadinoxanthin which determined higher ROS concentration compared to the other sixteen pigments (at least *p* < 0.01; Figure 2). Conversely, the carotenoids violaxanthin, astaxanthin and diatoxanthin were the best performing pigments in lowering intracellular ROS (145 nmol L^−1^, 130 nmol L^−1^, and 110 nmol L^−1^, respectively), although the results are not significantly different from those obtained with the other twelve pigments (*p* > 0.05).

Intracellular ROS concentration of B16-F0 cells exposed to UV-light. Values are expressed as mean ± SD of ROS nmol L^−1^. All pigments showed significant differences compared to control (no pigments with UV exposure; *p* ≤ 0.0001; Dunnet’s test). Ctr: control.

### 2.4. NO Concentration

**Figure 3:**
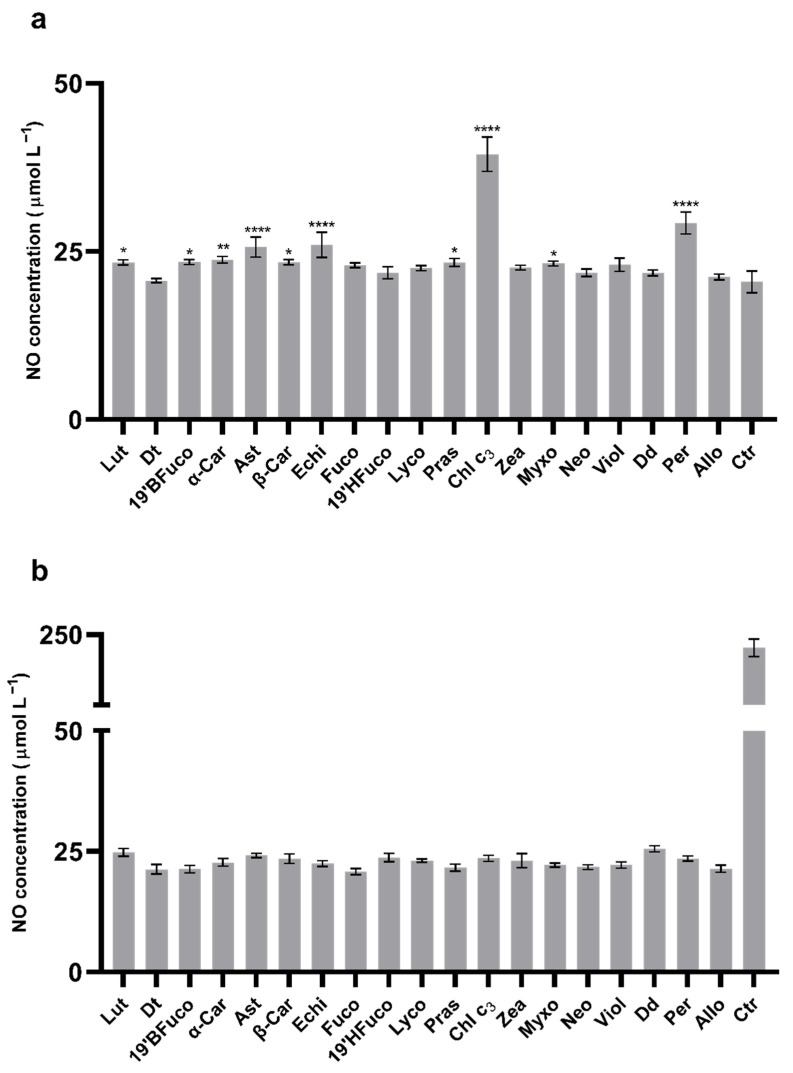
Extracellular NO measurements. (**a**) B16-F0 extracellular NO concentration without UV-radiations. Asterisks indicate the statistically significant differences compared to the control (23 μmol L^−1^; no pigment; **** *p*-value ≤ 0.0001, ** *p*-value ≤ 0.01, * *p*-value ≤ 0.05; Dunnett’s test). (**b**) B16-F0 extracellular NO concentration after 15 min UV-radiations. All pigments showed significant differences compared to the control (no pigments with UV exposure, *p*-value ≤ 0.0001; Dunnet’s test). All values are expressed as mean ± SD of NO μmol L^−1^. Ctr: control.

NO release by cells is one of the key processes in the inflammatory pathway onset related to ROS generation. It has been measured in presence or absence of pigments after UV exposure. Some pigments, such as lutein, 19′-butanoyloxyfucoxanthin, α-carotene, astaxanthin, β-carotene, echinenone, prasinoxanthin, chlorophyll c_3_, myxoxanthophyll, and peridinin, significantly increased the extracellular NO production compared to the control without pigment (23 μmol L^−1^; *p* < 0.05; Figure 3a). Conversely, diatoxanthin, fucoxanthin, 19′-hexanoyloxyfucoxanthin, lycopene, zeaxanthin, neoxanthin, violaxanthin, diadinoxanthin, and alloxanthin did not induce an increase of the NO release by the B16-F0 cells compared to the control (no pigment, *p* > 0.05; Figure 3a).

When the B16-F0 cells were exposed to UV radiations, the release of NO strongly increased (250 μmol L^−1^ with UV versus 23 μmol L^−1^ without UV). Pigments were able to significantly lower this increase (Figure 3b; *p* < 0.0001), with NO concentration ~ 25 μmol L^−1^, close to the value of the control without UV radiations (23 μmol L^−1^).

The link between NO release and ROS production when cells were treated with UV and pigments was confirmed with the linear and significant relationship between ROS and NO concentrations (*p* < 0.01).

### 2.5. Interleukin-1β (IL-1β) Concentration

**Figure 4:**
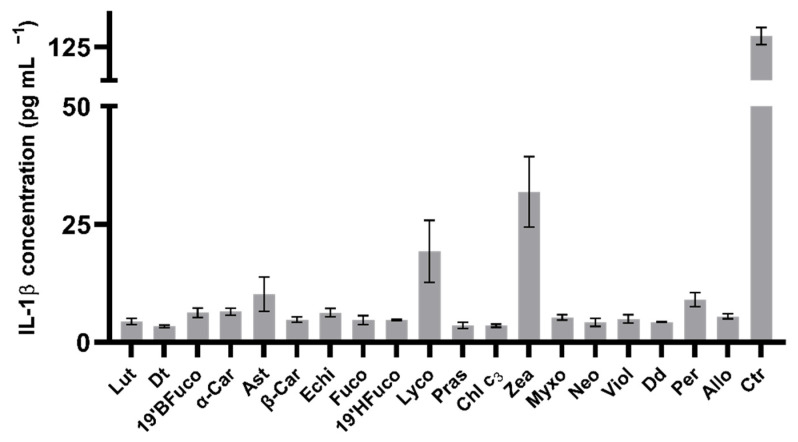
Extracellular IL-1 β levels.

One of the primary key factors participating to cytokine storming in the inflamed cells is the release of IL-1β. Independently on UV exposure, all pigments decreased the production of IL-1β compared to the control (without pigment nor UV: 21 pg mL^−1^; at least *p* < 0.05; data not shown). When cells were exposed to UV radiation, all pigments were able to lower the IL-1β production compared to the control which released 135 pg mL^−1^ of this cytokine in the medium (*p* < 0.0001; Figure 4), reaching values below 10 pg mL^−1^, except for lycopene (19 pg mL^−1^) or zeaxanthin (32 pg mL^−1^), with values significantly higher than for the other pigments (*p* < 0.01 and *p* < 0.0001, respectively).

Extracellular IL-1β concentration of B16-F0 cells exposed to UV light. Values are expressed as mean ± SD of IL-1β (pg mL^−1^). All pigments displayed significant differences compared to the control (no pigment with UV exposure, *p* ≤ 0.0001; Dunnet’s test). Ctr: control.

### 2.6. Matrix Metalloproteinase 9 Protein (MMP-9) Production

In order to assess the potential chemopreventive activity of pigments, MMP9 levels were measured by western blot analysis in pigment-treated or untreated cells after UV exposure.

**Figure 5:**
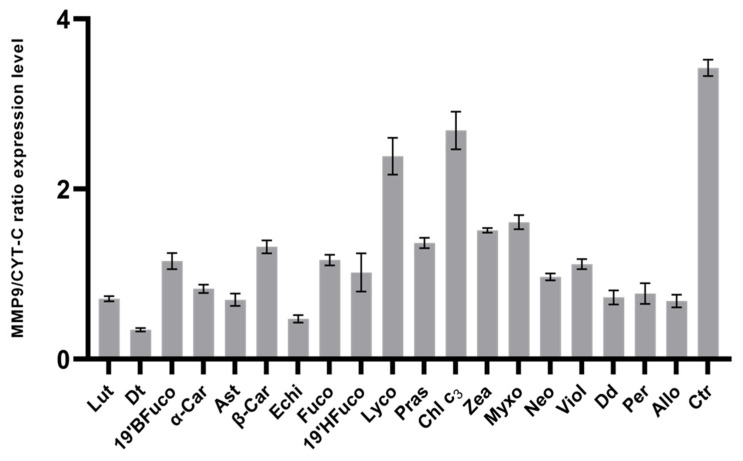
MMP-9 expression levels.

All pigments decreased MMP-9 production compared to the control (UV without pigment; Figure 5; *p* < 0.001), notwithstanding the high variability amongst pigments. Diatoxanthin determined a significantly lower production of MMP-9 compared to the other pigments (at least *p* < 0.05; Figure 5), resulting the best performing pigment in lowering MMP-9 levels. Conversely, chlorophyll c_3_ and lycopene were less performing, showing significantly higher level of MMP-9 production compared to all other pigments (Figure 5; *p* < 0.0001).

**Figure 6:**
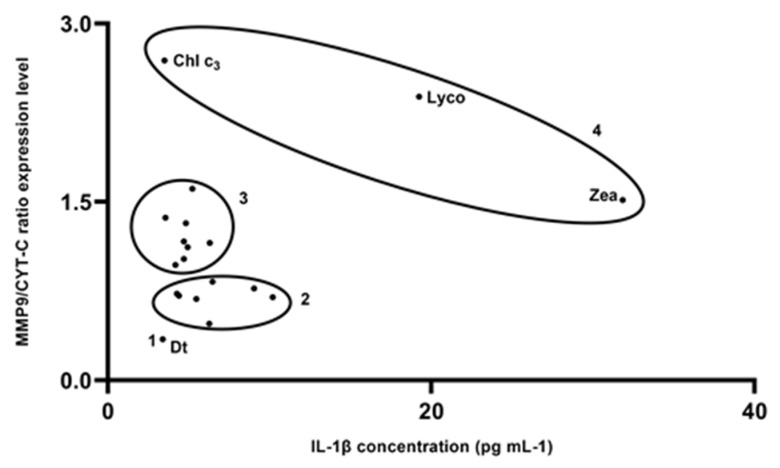
Efficacy of pigments in coping with IL-1β and MMP-9 cells production in UV-exposed cells. Scatter plot of MMP9/CYT-C ratio expression level versus extracellular IL-1β concentration in B16-F0 cells exposed to UV-light. Numbers refer to clusters described in the text.

In comparing the efficacy of pigments in coping with IL-1β and MMP-9 production in cells exposed to UV radiations (Figure 6), the first observation was the absence of any significant correlation (*p* > 0.05) between the two activities, highlighting the pigment-mediated independence between the two signals. In addition, four clusters were discriminated (Figure 6). Diatoxanthin was able to strongly lower both MMP-9 and IL-1β. A second group composed by the seven carotenoids echinenone, astaxanthin, diadinoxanthin, peridinin, alloxanthin, lutein, and α-carotene induced a small increase in MMP-9 synthesis, keeping a low IL-1β secretion. The less efficient group was composed by the two carotenoids lycopene and zeaxanthin and the chlorophyll c_3_. The other eight pigments (19′BFuco, astaxanthin, 19′HFuco, fucoxanthin, prasinoxanthin, neoxanthin, violaxanthin, β-carotene) belonged to the intermediary third group (Figure 6). Intriguingly, comparing the intracellular MMP-9 response with the pigment structure (Supplementary File S1), it was noteworthy that MMP-9 production was significantly lowered (*p* < 0.003) by carotenoids with H/C (hydrogen/carbon) ratio ≤ 1.35 (0.62 ± 0.16, n = 6) compared to carotenoids with H/C ratio > 1.35 (1.26 ± 0.44, n = 12).

Mean ± SD of the ratio between the expression of matrix metallopeptidase 9 and cytochrome C (used as positive control) retrieved from western blot analysis (see Section 4.11). Densitometric analysis of immunopositive bands was performed using ImageLab software.

All pigments showed significant differences compared to the control (no pigments with UV exposure; *p* ≤ 0.0001; Dunnet’s test). Note that the pigment ordering is different between panels a and b. Ctr: control.

## 3. Discussion

The bioactivity exploration of natural antioxidant can lead to numerous human health application, as, for instance, in preventing or healing cellular damages caused by UVs. In this context, compounds able to exert photochemopreventive activities are significant targets [31,32,33], since photochemoprevention might represent a valuable solution to prevent or limit the occurrence and the severity of UV-related diseases and photocarcinogenesis. The latter is linked to the multiple effects of UV radiation on skin [34], including photoaging, release of reactive oxygen species, DNA mutations, inflammation, and release of immunomodulatory cytokines. Efficacy of photochemopreventive effects depends on different factors, such as the ability of the molecule to absorb UVA and UVB rays [35], the antioxidant effect of the molecule and ROS scavenging, the inhibition of MMPs, which could damage or destroy the collagen and elastic fibers that constitute the dermis, and the modulation of stress-dependent signaling and/or suppression of cellular and tissue responses, such as inflammation and cytokines release [35,36]. The searching and discovery of natural photochemopreventive products requires investigations on the molecular mechanisms, to evaluate their activities and effects on cell signaling processes caused by UV exposure. Although pigments are receiving a growing interest for their bioactivity (e.g., Reference [11]), only few of them are known, and, in this context, an important gap of knowledge is remains to be filled. The unbalance between, on one hand, the opportunities and challenges offered by microalgae in a biotechnological context [21,37] and, on the other hand, the lack of knowledge of the biological properties towards human health benefits (e.g., References [22,23]) represents a limit for the further industrial blue biotechnology development.

This study investigates the photochemopreventive activity of twenty microalgal pigments, using the murine B16-F0 cell line model. In this model, the angiogenesis and invasiveness properties are constitutively activated by upregulation of MMP-9 [30,38]. Among the different interconnected parameters analyzed, the investigation of MMP inhibition can be related to the evaluation of chemopreventive and antimetastatic effects [39].

The comparative analysis highlights that not all pigments do act in the same way or with the same extent towards cell signaling and inflammation processes involved in photocarcinogenesis. Pigment molecular structure or composition does not explain the observed results’ variability, e.g., on the DPPH scavenging or lowering ROS concentration, as it was hypothesized [40]. Variability in those capacities might rely on the properties of the carotenoid radicals formed after the radical scavenging [41]. The antioxidant and anti-inflammatory activities are generally linked to each other [42]: the increased antioxidant capacity inhibits the inflammatory signal transduction through a reduced release of nitric oxide (NO) and IL-1β [43,44]. However, in this study, the different pigment activities explored (DPPH scavenging, ROS, NO, IL-1β, or MMP-9 lowering) are not directly—or little—linked to one another. The high DPPH scavenging ability of lutein, diatoxanthin, 19′-butanoyloxyfucoxanthin, and α-carotene is paralleled by their great capacity of lowering intracellular ROS concentrations in cells experiencing UV radiations. The two scavenging activities are positively correlated (*p* < 0.05), excluding three pigments: diadinoxanthin, 19′-hexanoyloxyfucoxanthin (displaying the highest ROS concentration), and alloxanthin (displaying the lowest DPPH scavenging value).

Most of the pigments (excluding zeaxanthin and lycopene) greatly lower inflammatory response by reducing the IL-1β release, but their effect on MMP-9 is more variable. The lycopene or chlorophyll c_3_ appear not efficient in coping with MMP-9 production. This indicates that some pigments exert partial photochemopreventive activity relied on ROS scavenging or anti-inflammatory properties, but they do not necessarily act on the MMP pathway. The best performing carotenoids in lowering MMP-9 and IL-1β are the exclusive aquatic carotenoids diatoxanthin, diadinoxanthin, peridinin, and alloxanthin, together with other carotenoids, i.e., astaxanthin, echinenone, lutein, and α-carotene. Bioactivity of peridinin and diatoxanthin were recently highlighted in inhibiting A2058 malignant melanoma cell line growth [45], and the same result was obtained with alloxanthin [46]. Results obtained in the present study underline that all microalgal groups display at least one of these compounds: lutein in green algae, alloxanthin in cryptophytes, peridinin in dinophytes, and diadinoxanthin-diatoxanthin in diatoms or other heterokontophyta. It is also noteworthy that the four exclusive aquatic carotenoids, diatoxanthin, diadinoxanthin, alloxanthin, or peridinin, belong to microalgal groups (diatoms, cryptophytes, and dinophytes) with a low number of carotenoid compounds, and three of them (diatoxanthin, diadinoxanthin, and alloxanthin) are involved in photoprotective responses [20]. Interestingly, these three are biosynthetically linked together [11,47] and belong to the less common acetylenic carotenoid group, already reported for its potential great bioactivity [48]. Diatoxanthin (Dt) addresses all the key steps requested for an efficient photochemopreventive activity: ROS scavenging, anti-inflammatory towards NO/IL-1β signaling, and antimetastatic through the inhibition of MMP-9 expression. Dt is a photoprotective pigment synthetized by microalgae under high light [16,20,25,49,50,51], or more generally involved in processes protecting microalgae from environmental stresses, e.g., nutrient depletion or viral attacks [20]. Alloxanthin is only present in Cryptophytes, which might be an interesting target in providing the anti-inflammatory bioactive molecule [46]. Interests in this microalgal group are also enhanced by its content in other pigments, such as the water soluble bioactive phycobiliproteins [52].

Conversely, peridinin, an uncommon C39 carotenoid, displays an allene structure [53], and it is the main carotenoid of some dinoflagellates, a group which encloses more than 90 toxigenic [54] over the 2000 described species. Intriguingly, peridinin is the only pigment displaying cytotoxic effect on the targeted cell line. In addition, while it shows a potential antiangiogenic effect by the inhibition of MMP-9 pathway in the premetastatic cell line, it is one of the less performing pigments in terms of DPPH scavenging, ROS, NO, and IL-1β production inhibition.

## 4. Materials and Methods

### 4.1. Investigation Strategy and Experimental Pipeline

**Figure 7:**
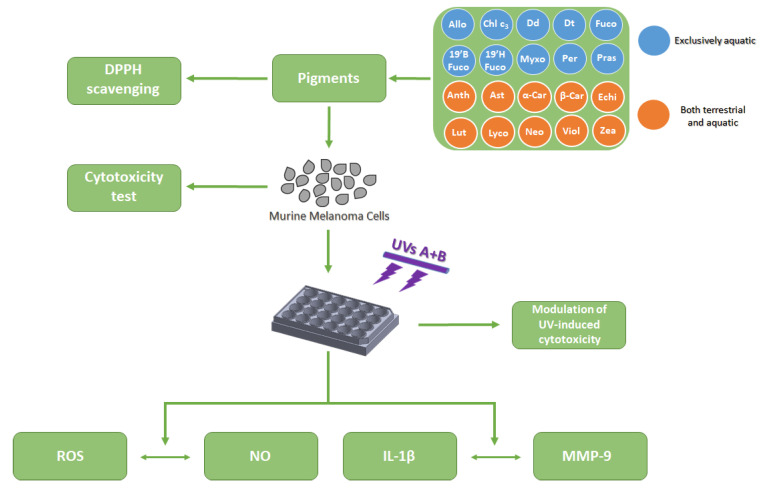
Experimental pipeline for the comparative analysis conducted. Abbreviations as in Table 1.

To assess microalgal pigments ability as photochemopreventive agent on the murine melanoma cell model B16-F0, we focused on their potential capability to inhibit the interleukin 1-β release and metalloproteinase-9 expression (Figure 7). The pigments’ ability to scavenge DPPH radical was assessed, allowing to determine the minimum effective concentration of pigment able to scavenge radical and to be used for the in vitro experiments. Once the in vitro cytotoxicity of pigments on the human cell line normal keratinocytes HaCaT and on the murine melanoma B16-F0 cell line was evaluated, the B16-F0 cell line was exposed to 15 min UVs radiation (λmax = 310 nm, range from 280 to 360 nm; see Section 4.6), with or without pigment previous addition. The photochemopreventive ability of pigments was then estimated through their capacity to counteract the UV-induced reactive oxygen species generation, nitric oxide release, as well as the interleukin 1-β and metalloproteinase-9 synthesis (Figure 7).

### 4.2. Microalgal Pigments

The twenty microalgal pigments were selected as follows: ten were exclusive of aquatic organisms, and ten were shared by both aquatic and terrestrial organisms (Table 1, Supplementary File S2). Nineteen pigments belonged to the family of carotenoids and one to the chlorophylls. The latter, chlorophyll c_3_, was selected following results demonstrating its intracellular concentration modulation in microalgae with light climate variations, in parallel with some carotenoids [27]. Among the selected carotenoids, some belonged to photoprotective pigment cycles (xanthophyll cycle, XC), while the others were photosynthetic (Table 1). All the pure pigments were purchased from the D.H.I. Water & Environment (Hørsholm, Denmark).

### 4.3. DPPH (2,2-Diphenyl-1-picrylhydrazyl) Assay

An aliquot of 2.5 μL of pigment-methanol solution at different concentrations—was mixed with 2.5 μL of 2,2-diphenyl-1-picrylhydrazyl (DPPH; CAS No. 1898-66-4, cat. No. 257621, Sigma-Aldrich, St. Louis, MO, USA) for a final concentration *w*/*v* of 25, 50, 125, 250, and 500 ng mL^−1^. DPPH was weighted and dissolved in methanol. The pigment-methanol solutions and DPPH were mixed in 0.2 mL tubes, with DPPH at a final concentration of 100 μM, and kept for 30 min in the dark. Methanol was used as negative control. After incubation, the absorbance of DPPH solution was measured at 517 nm, using a Microplate Reader: Infinite^®^ M1000 PRO (TECAN, Männedorf, Switzerland). Methanol absorbance was subtracted from sample and control absorbances. The scavenging assay was performed in triplicate. The results were expressed as percentage reduction of the initial DPPH absorption in relation to the control (methanol solution), calculated according to the equation: DPPH scavenging effect (%)/% Inhibition = A_0_ − (A_1_/A_0_) × 100, where A_0_ = absorbance of control (Trolox solution) and A_1_ = absorbance of sample.

The DPPH assay was performed in 0.2 mL tubes after validation (Supplementary File S3), through a comparative analysis of the antioxidant activity of (±)-6 hydroxy-2,5,7,8-tetramethylchromane-2-carboxylic acid (Trolox; CAS No. 53188071, cat. No. 238813, Sigma-Aldrich, St. Louis, MO, USA) in 96-well plate (i.e., following the traditional protocol consisting in mixing 90 µL of Trolox with 90 µL of DPPH solution 0.1 M) [55] versus the 0.2 mL tube. Trolox powder was weighted and dissolved in methanol at five concentrations: 0.1–1–10–100–1000 µg mL^−1^.

### 4.4. The Human HaCaT Keratinocytes Cell Line

HaCaT is a spontaneously transformed aneuploid immortal keratinocyte cell line from adult human skin which represents a reproducible model for the characterization of several processes, such as cytotoxicity of substances suitable for topic use. The HaCaT cell line was kindly provided by the Human and Animal Cell Lines supply from CEINGE-Biotecnologie Avanzate research Institute (Naples). HaCaT cells were grown in DMEM-F12 supplemented with 10% (*v*/*v*) fetal bovine serum (FBS), 100 units ml^−1^ penicillin, 100 units ml^−1^ streptomycin, and 2 mM of L-glutamine, in a 5% CO_2_ atmosphere at 37 °C. HaCaT cells (2 × 10^5^ cells ml^−1^) were seeded in a 96-well plate (TPP, Techno Plastic Products AG, Trasadingen, Switzerland) and kept overnight for attachment. All experiments were performed in triplicate.

### 4.5. The Murine B16-F0 Melanoma Cell Line

The primary murine B16-F0 melanoma cell line is a well-established model system for studying many aspects of cancer biology, including metastasis [26,56]. Moreover, this metastatic cell line was already used for studying MMP-9 expression [26]. The murine B16-F0 melanoma line was purchased from the ATCC (product code: ATCC^®^ CRL-6322™) and grown in DMEM-F12 supplemented with 10% (*v*/*v*) FBS, 100 units ml^−1^ penicillin, 100 units ml-1 streptomycin, and 2 mM of L-glutamine, in a 5% CO_2_ atmosphere at 37 °C. B16-F0 cells (2 × 10^5^ cells ml^−1^) were seeded in a 24-well plate (TPP, Techno Plastic Products AG, Trasadingen, Switzerland) and kept overnight for attachment. All experiments were performed in triplicate.

### 4.6. UV Radiations and Pigment Effect Assessment

The 24-well plates containing the B16-F0 cells were transferred under UVs radiation for 15 min in an axenic environment with the same growth conditions as described before. The UV radiation was provided by a Philips UVB neon tube (TL 20W/12 RS SLV/25; λmax = 310 nm, range from 280 to 360 nm). The plates containing murine cells were placed at 15 cm from the UVs source, and the intensity experienced by cells was 5.5 mW cm^−2^ of UV-B (280–315 nm) and 0.08 mW cm^−2^ of UV-A (315–400 nm).

To assess the role of pigments in protecting or inhibiting the UV damage effects on the B16-F0 mouse cell line, the latter was previously treated for 1 h with pigments at a concentration of 25 ng ml^−1^ (see DPPH results section) before UV exposure.

### 4.7. Cell Viability Assessment

Murine B16-F0 and human HaCaT cells viability was assessed using the MTT (3-(4,5-Dimethylthiazol-2-yl)-2,5-Diphenyltetrazolium Bromide, Applichem, Darmstadt, Germany) assay. Potential pigment cytotoxicity was evaluated on cells pre-treated for 48 h with pigments at a concentration of 25 ng mL^−1^ and compared with untreated cells. In addition, B16-F0 cell viability was assessed after the 15-min UV exposure on both pre- and untreated cells to assess pigments’ protective effect. At the end of the different treatments, cells were incubated with 10 µL (5 mg mL^−1^) of MTT and incubated for 3 h at 37 °C with 5% CO_2_. The resulting formazan crystals only produced by viable cells were dissolved with 100 µL of isopropyl alcohol. The absorbance was recorded using a Microplate Reader: Infinite^®^ M1000 PRO (TECAN, Männedorf, Switzerland) at a wavelength of 570 nm.

### 4.8. Intracellular Reactive Oxygen Species (ROS) Measurement

Reactive Oxygen Species (ROS) intracellular levels were measured using the OxiSelect™ Intracellular ROS Assay Kit (cat. No. STA-342, Cell Biolabs, Inc., San Diego, CA, USA), according to manufacture protocol. After the treatments—without versus with pigments at a concentration of 25 ng ml^−1^ and without versus with 15-min UV radiations—the medium was removed from the 24-well plate, and a mixture of DMEM-F12 and 2X Cell Lysis Buffer (supplied in the kit) was added to each well. Cells were incubated with Dichloro-dihydro-fluorescein diacetate (DCFH-DA, supplied in the kit) for 1 h. Fluorescence was then read using a Microplate Reader: Infinite^®^ M1000 PRO (Ex: 480 nm, Em: 530 nm, TECAN, Männedorf, Switzerland). ROS concentration was quantified to a calibration curve obtained by measuring fluorescence of different concentrations of 2′,7′-Dichlorodihydrofluorescein (DCF, supplied in the kit). Intracellular ROS concentration (nM) was compared amongst the different treatments, assessing the role of UV and pigments in modulating it.

### 4.9. Extracellular Nitric Oxide (NO) Measurement

Quantification of NO was performed using a Colorimetric Assay Kit (cat. No. E-BC-K035-M, Elabscience, Houston, TX, USA). After the treatments—without versus with pigments at a concentration of 25 ng ml^−1^ and without versus with 15-min UV radiations—the medium was collected. Collected medium was then used to assess NO concentration, according to manufacture protocol. The absorbance of medium was then measured at 550 nm using a Microplate Reader: Infinite^®^ M1000 PRO (TECAN, Männedorf, Switzerland). The NO concentration was quantified to a calibration curve obtained by measuring the absorbance of different concentrations of a sodium nitrite standard solution (supplied in the kit). The assay was performed in triplicate. NO concentration (µMol L^−1^) in the medium was compared amongst the different treatments, assessing the role of UV and pigments in modulating it.

### 4.10. Interleukin 1 β (IL-1β) Measurement

Quantification of IL-1β was performed using an ELISA Kit (Cat. BMS224-2; Invitrogen, Carlsbad, CA, USA). After the treatments—without versus with pigments at a concentration of 25 ng ml^−1^ and without versus with 15-min UV radiations—the medium was collected and used to assess the release of IL-1β from cells, according to manufacturing protocol. The absorbance was measured at 450 nm using a Microplate Reader: Infinite^®^ M1000 PRO (TECAN, Männedorf, Switzerland). IL-1β concentration in the medium was quantified to a calibration curve obtained by measuring the absorbance of different concentrations of an IL-1β solution (supplied in the kit). ELISA experiments were performed in triplicate. IL-1β concentration (pg mL^−1^) in the medium was compared amongst the different treatments, assessing the role of UV and pigments in modulating it.

### 4.11. Matrix Metalloproteinase 9 Protein (MMP-9) Measurement

Production of MMP-9 was assessed performing a western blot analysis on cells pre- and untreated with pigments and exposed to UV radiation. The medium was removed, while the cell lysates were prepared by scraping each well into 500 µL of RIPA Lysis and Extraction Buffer (Thermo Fisher Scientific, Waltham, MA, USA), supplemented with Halt™ Protease & Phosphatase Inhibitor Cocktail (Thermo Fisher Scientific, Waltham, MA, USA). The lysate was incubated on ice for 15 min and then clarified by centrifugation at 14,000 g for 20 min. Total protein concentration was determined according to the Bradford method using Bradford—Solution for Protein Determination (cat. No. A6932, Applichem, Darmstadt, Germany) with bovine serum albumin (BSA, cat. A2058, Sigma-Aldrich, St. Louis, MO, USA) as a standard. The protein extracts were stored at −20 °C until use. Before electrophoresis, protein samples were incubated at 95 °C for 5 min. Following that, 10% SDS-PAGEs were stained with Coomassie Brilliant Blue R-250 Staining Solution (cat. No. 161-0436, Bio-Rad, Hercules, CA, USA) or blotted onto Trans-Blot Turbo Midi 0.2 µm Nitrocellulose membrane (cat. No. 170-4159, Bio-Rad, Hercules, CA, USA) using Trans-Blot Turbo Transfer System (cat. No. 170-4150, Bio-Rad, Hercules, CA, USA). Membranes were incubated for 1 h in blocking reagent (1X Tris Buffered Saline-TBS), with 0.1% Tween-20 with 5% *w*/*v* nonfat dry milk, and incubated overnight at 4 °C with the primary antibodies diluted in 1X TBS, 0.1% Tween-20 with 5% BSA. MMP-9 protein was investigated (antibody 1:1000 diluted, ab38898, Abcam, Darmstadt, Germany). Positive control was obtained by using an anti-cytochrome complex antibody (CYT-C, 1:5000 diluted, ab90529, Abcam, Darmstadt, Germany). After incubation, membranes were washed three times for 10 min each with 15 mL of TBS/Tween and then incubated with Goat Anti-Rabbit IgG H&L Alexa Fluor^®^ 488 (1:2000 dilution, ab150077, Abcam, Darmstadt, Germany) with gentle agitation for 1 h at room temperature. After incubation, membranes were washed three times for 5 min each with 15 mL of TBS/Tween. Blotted membranes were detected by visualizing proteins with ChemiDoc™ MP Imaging System (cat. No. 120-3154, Bio-Rad, Hercules, CA, USA). Densitometric analysis of immunopositive bands was performed using ImageLab software (Bio-Rad, Hercules, CA, USA).

### 4.12. Data Treatment and Statistical Analysis

All statistical analyses were performed by using GraphPad^®^ Software. Mean and standard deviations from the triplicate analysis for each parameter was calculated. Spearman correlation was performed. Mean comparison was carried out using one-way ANOVA to assess the variance within the control and treated groups and between these experimental groups. A different analysis was made for DPPH assay, in which 2-way ANOVA was used. Dunnett’s method was applied to compare the mean from treated groups with the control mean (untreated cells), while Tukey’s method was applied to compare the mean from each treated group with every other treated group.

## 5. Conclusions

Specific aquatic carotenoids belonging to microalgae, such as diatoxanthin, diadinoxanthin, alloxanthin, or peridinin, display interesting photochemopreventive activity in being able to lower intracellular oxidative stress and inflammation signaling induced by UV stress. This study highlights, for the first time, the bioactivity of diatoxanthin, suggesting to invest research efforts on this molecule, and, thus, on diatoms—known to synthetize it—, for biotechnological applications. Cryptophytes, with their unique alloxanthin, are also relevant alternative candidates in this context. This study emphasizes the positive outcomes of a deeper integration of plant ecophysiological knowledge into the development of bioprospecting pipeline aiming to discover and exploit new molecules with human health benefits for biotechnological applications, such as in nutraceuticals or cosmeceuticals.

## Figures and Tables

**Table 1:** List of the twenty algal pigments.

ID	Pigment	Microalgal Group (s)	Aquatic Specific or Not?	Roles	Chemical STRUCTURE	H/C Ratio
*Allo*	Alloxanthin	Cryptophytes	Yes	Photosynthetic	C_40_H_52_O_2_	1.30
*Anth*	Antheraxanthin	Green algae	No	Photoprotective, XC	C_40_H_56_O_3_	1.40
*Ast*	Astaxanthin	Some chlorophytes	No	environmental stress response	C_40_H_52_O_4_	1.30
*α-Car*	α-carotene	Green algae	No	Photosynthetic	C_40_H_56_	1.40
*β-Car*	β-carotene	all	No	Photosynthetic	C_40_H_56_	1.40
*Chl c_3_*	Chlorophyll c_3_	Mainly haptophytes	Yes	Photosynthetic	C_36_H_28_MgN_4_O_7_	0.80
*Dd*	Diadinoxanthin	Mainly: diatoms, xanthophytes, haptophytes, dinophytes	Yes	Photosynthetic, XC	C_40_H_54_O_3_	1.35
*Dt*	Diatoxanthin	Mainly: diatoms, xanthophytes, haptophytes, dinophytes	Yes	Photoprotective, XC	C_40_H_54_O_2_	1.35
*Echi*	Echinenone	Some cyanophytes	No	Photosynthetic	C_40_H_54_O	1.35
*Fuco*	Fucoxanthin	Mainly diatoms	Yes	Photosynthetic	C_42_H_58_O_6_	1.38
*19′* *BFuco*	19′-butanoyloxy-fucoxanthin	Pelagophytes/Crysophytes	Yes	Photosynthetic	C_46_H_64_O_8_	1.39
*19′* *HFuco*	19′-hexanoyloxy-fucoxanthin	Mainly haptophytes	Yes	Photosynthetic	C_48_H_68_O_8_	1.42
*Lut*	Lutein	Green algae	No	Photosynthetic, Photoprotective	C_40_H_56_O_2_	1.40
*Lyco*	Lycopene	All	No	Precursor of β-carotene	C_40_H_56_	1.40
*Myxo*	Myxoxanthophyll	Cyanophytes	Yes	Structural role	C_46_H_66_O_7_	1.43
*Neo*	Neoxanthin	Green algae	No	Photosynthetic, Photoprotective	C_40_H_56_O_4_	1.40
*Per*	Peridinin	Dinophytes	Yes	Photosynthetic	C_39_H_50_O_7_	1.28
*Pras*	Prasinoxanthin	Some prasinophytes	Yes	Photosynthetic	C_40_H_56_O_4_	1.40
*Viol*	Violaxanthin	Green algae	No	Photosynthetic, XC	C_40_H_56_O_4_	1.40
*Zea*	Zeaxanthin	Green algae; cyanophytes	No	Photoprotective XC; photosynthetic (cyano)	C_40_H_56_O_2_	1.40

XC: xanthophyll cycle; cyano: cyanophytes; H/C ratio: Hydrogen/Carbon ratio.

**Table 2:** DPPH Radical scavenging capacity of the twenty pigments at five concentrations. Values are expressed as mean ± SD of the percentage of DPPH inhibition. Pigments were ordered from the highest to the lowest scavenging activity measured at 25 ng mL^−1^. Abbreviations as in Table 1.

	25 ng mL^−1^	50 ng mL^−1^	125 ng mL^−1^	250 ng mL^−1^	500 ng mL^−1^
*Lut*	48.80 (±0.85)	46.39 (±0.85)	47.59 (±0.85)	−15.06 (±4.26)	−12.65 (±0.85)
*Dt*	46.99 (±9.56)	39.36 (±9.81)	37.35 (±6.26)	24.50 (±8.20)	19.68 (±1.39)
*19′BFuco*	46.18 (±3.03)	50.20 (±5.94)	46.99 (±1.20)	55.42 (±12.58)	72.69 (±1.39)
*α-Car*	45.89 (±1.36)	34.62 (±8.98)	43.96 (±8.42)	46.86 (±5.88)	20.45 (±0.56)
*Ast*	43.64 (±3.90)	18.20 (±1.12)	31.08 (±5.82)	32.69 (±4.02)	19.16 (±5.66)
*β-Car*	42.35 (±3.11)	45.57 (±7.50)	37.20 (±6.69)	43.32 (±17.29)	39.13 (±7.55)
*Echi*	38.55 (±5.25)	27.31 (±5.94)	22.49 (±3.87)	35.54 (±2.56)	42.77 (±7.67)
*Fuco*	36.14 (±11.04)	40.36 (±9.37)	36.55 (±9.74)	45.38 (±13.22)	58.43 (±0.85)
*19′HFuco*	36.14 (±8.52)	39.36 (±11.70)	41.37 (±9.66)	42.17 (±2.41)	60.24 (±4.34)
*Anth*	25.60 (±2.73)	35.91 (±18.97)	49.76 (±8.20)	58.94 (±7.52)	62.80 (±0.68)
*Lyco*	21.69 (±8.52)	33.73 (±8.52)	36.55 (±12.07)	54.22 (±8.35)	62.01 (±17.84)
*Pras*	17.53 (±1.69)	17.53 (±3.38)	17.53 (±4.78)	24.10 (±2.54)	29.48 (±2.39)
*Chl c_3_*	17.39 (±6.15)	31.88 (±8.88)	35.27 (±1.37)	40.74 (±5.90)	43.48 (±3.42)
*Zea*	17.13 (±0.69)	19.40 (±0.61)	23.21 (±1.27)	26.49 (±0.85)	45.62 (±4.23)
*Myxo*	17.13 (±3.01)	16.93 (±0.85)	15.14 (±1.69)	15.14 (±17.69)	22.91 (±0.85)
*Neo*	16.33 (±1.20)	15.74 (±0.85)	22.91 (±2.54)	23.11 (±1.38)	26.69 (±6.58)
*Viol*	14.54 (±4.23)	16.33 (±6.76)	19.32 (±17.75)	19.74 (±0.25)	21.51 (±3.65)
*Dd*	13.86 (±0.85)	18.47 (±4.56)	20.48 (±2.41)	26.10 (±0.70)	25.70 (±2.78)
*Per*	2.59 (±0.85)	7.17 (±1.83)	19.02 (±0.42)	20.52 (±0.85)	25.30 (±5.92)
*Allo*	0.97 (±0.68)	10.14 (±1.37)	28.50 (±12.30)	42.03 (±6.83)	61.35 (±4.10)

## Data Availability

The data presented in this study are available on request from the corresponding author.

## Supplementary Materials

The following are available online at https://www.mdpi.com/article/10.3390/md19070354/s1, Supplementary File S1: Title of data: Relation between MMP-response and pigment structure. Description of data: MMP-9 response of pigments was compared by relating it to their chemical structure, in terms of H/C ratio. Supplementary File S2: Title of data: Chemical structure of the twenty pigments used in this study Supplementary File S3: File format: .docx Title of data: Validation of DPPH assay protocol in 0.2 mL Eppendorf. Description of data: (A) DPPH assay results obtained following the traditional protocol in 96-well plate. (B) DPPH assay results obtained following the modified protocol in 0.2 mL eppendorf. On the left, DPPH inhibition, expressed in percentage, of increasing Trolox concentrations (0.1, 1, 10, 100, and 1000 µg/mL). The results are expressed as percent of DPPH inhibition calculated as the ratio between mean absorbance of each treatment and mean absorbance of control. Asterisks indicate the statistically significant difference compared to a negative control for * *p* < 0.05, ** *p* < 0.01, *** *p* < 0.0001 (Dunnett’s test). On the right, Dose-response curve of increasing Trolox concentrations, expressed in percentage, in DPPH assay. All values are represented as the means S.D. of three independent experiments.
