# Supplementary material for: MMP-9 and IL-1β as Targets for Diatoxanthin and Related Microalgal Pigments: Potential Chemopreventive and Photoprotective Agents
Source: Mar Drugs. 2021 Jun 22;19(7):354. doi: 10.3390/md19070354 (PMC8303339; doi:10.3390/md19070354)

Alloxanthin

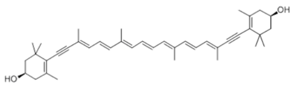

Antheraxanthin

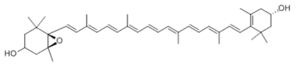

Astaxanthin

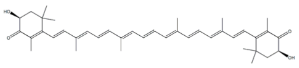

α-carotene

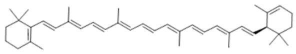

β-carotene

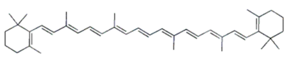

Chlorophyll c3

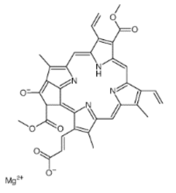

Diadinoxanthin

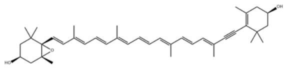

Diatoxanthin

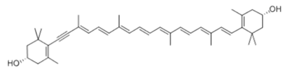

Echinenone

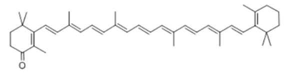

Fucoxanthin

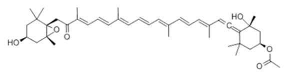

19’butayloxy-
fucoxanthin

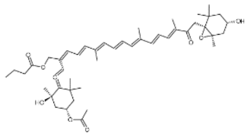

19’hexanoyloxy-
fucoxanthin

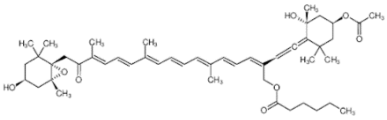

Lutein

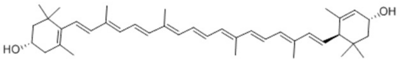

Lycopene

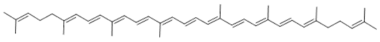

Myxoxanthophyll

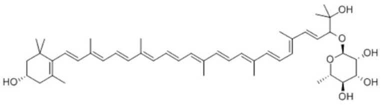

Neoxanthin

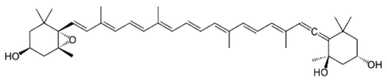

Peridinin

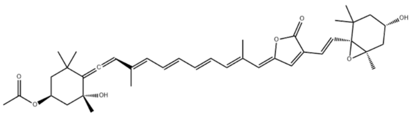

Prasinoxanthin

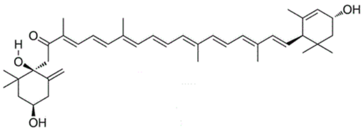

Violaxanthin

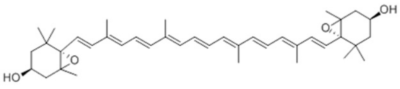

Zeaxanthin

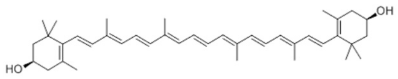

Supplement: Supplementary file 1 [file marinedrugs-19-00354-s001.zip › marinedrugs-1259938-supplementary/Additional file 2_Pistelli et al..pdf]
