# Supplementary figures and images for: MMP-9 and IL-1β as Targets for Diatoxanthin and Related Microalgal Pigments: Potential Chemopreventive and Photoprotective Agents
Source: Mar Drugs. 2021 Jun 22;19(7):354. doi: 10.3390/md19070354 (PMC8303339; doi:10.3390/md19070354)

Additional file 1

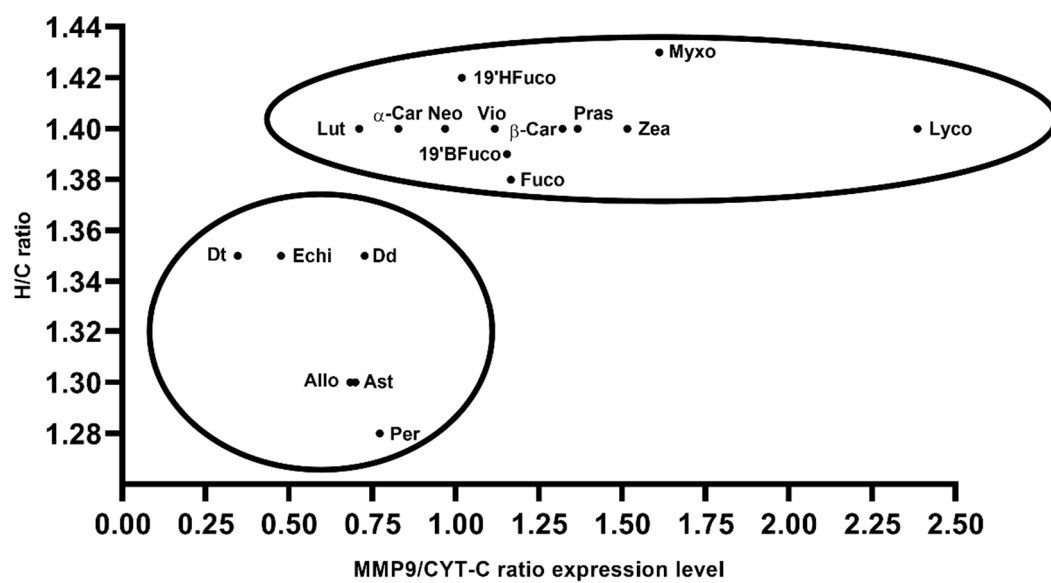

Supplement: Supplementary file 1 [file marinedrugs-19-00354-s001.zip › marinedrugs-1259938-supplementary/Additional file 1_pistelli et al.pdf]

### Additional file 3

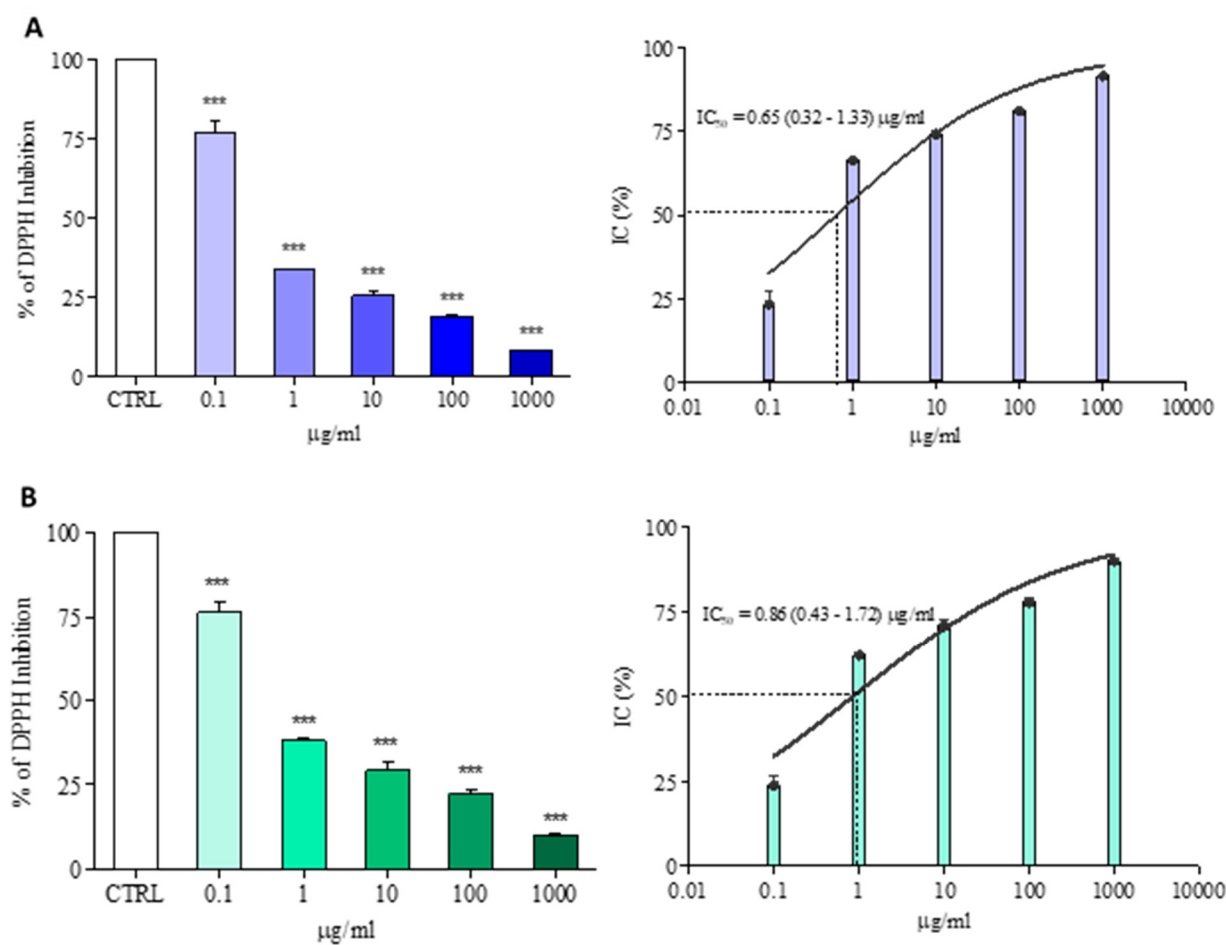

Supplement: Supplementary file 1 [file marinedrugs-19-00354-s001.zip › marinedrugs-1259938-supplementary/Additional file 3_pistelli et al.pdf]
